# Bipartite *rgp* Locus Diversity in Streptococcus thermophilus Corresponds to Backbone and Side Chain Differences of Its Rhamnose-Containing Cell Wall Polysaccharide

**DOI:** 10.1128/aem.01504-22

**Published:** 2022-11-09

**Authors:** Katherine Lavelle, Irina Sadovskaya, Evgeny Vinogradov, Philip Kelleher, Gabriele A. Lugli, Marco Ventura, Douwe van Sinderen, Jennifer Mahony

**Affiliations:** a School of Microbiology and APC Microbiome Ireland, University College Cork, Cork, Ireland; b Université du Littoral Côte d'Opale, Boulogne-sur-Mer, France; c National Research Council Canada, Ottawa, Ontario, Canada; d Laboratory of Probiogenomics, Department of Chemistry, Life Sciences and Environmental Sustainability, University of Parma, Parma, Italy; e Microbiome Research Hub, University of Parma, Parma, Italy; University of Naples Federico II

**Keywords:** Rgp structure, *rgp* loci, genetic variation, strain characterization

## Abstract

The rhamnose-glucose polysaccharide (Rgp) of Streptococcus thermophilus represents a major cell wall component, and the gene cluster responsible for its biosynthesis (termed *rgp*) has recently been identified. Significant genetic diversity among these loci has previously been reported, with five distinct *rgp* genotypes identified (designated *rgp1* through -*5*). In the present study, two additional genotypes were identified (designated *rgp6* and *rgp7*) through comparative analysis of the *rgp* loci of 78 Streptococcus thermophilus genomes. The *rgp* locus of a given S. thermophilus strain encoded the biosynthetic machinery for a rhamnan-rich backbone and a variable side chain component, the latter being associated with the highly specific interactions with many bacteriophages that infect this species. The chemical structure of the Rgp from three S. thermophilus strains, representing the *rgp2*, *-3*, and -4 genotypes, was elucidated, and based on bioinformatic and biochemical analyses we propose a model for Rgp biosynthesis in dairy streptococci. Furthermore, we exploited the genetic diversity within the S. thermophilus bipartite *rgp* locus to develop a two-step multiplex PCR system to classify strains based on gene content associated with the biosynthesis of the variable side chain structure as well as the rhamnan backbone.

**IMPORTANCE** Streptococcus thermophilus is present and applied in industrial and artisanal dairy fermentations for the production of various cheeses and yogurt. During these fermentations, S. thermophilus is vulnerable to phage predation, and recent studies have identified the rhamnose-glucose polymer (Rgp) as the definitive receptor for at least one problematic phage species. Detailed analysis of S. thermophilus *rgp* loci has revealed an unprecedented level of genetic diversity, particularly within the glycosyltransferase-encoding gene content of a given locus. Our study shows that this genetic diversity reflects the biochemical structure(s) of S. thermophilus Rgp. As such, we harnessed the genetic diversity of S. thermophilus *rgp* loci to develop a two-step multiplex PCR method for the classification of strain collections and, ultimately, the formation of phage-robust rational starter sets.

## INTRODUCTION

The biosynthesis and chemical structure of rhamnose-containing cell wall polysaccharides (CWPS) of ovococcal Gram-positive bacteria have been described in detail for pathogenic streptococci, enterococci, and lactococci (1–5). Unlike exopolysaccharides (EPS), which may be loosely bound to the cell surface and released to the environment, rhamnose-containing CWPS are covalently bound to the peptidoglycan layer and are known to play a role in virulence, immune modulation, cellular morphology, and phage attachment (1, 2).

The genomic locus which encodes the rhamnose-CWPS biosynthetic machinery often displays a modular arrangement with distinct conserved and variable regions. The conserved regions encode functions associated with rhamnan backbone synthesis, including the well-characterized rhamnosyltransferases RgpA, RgpB, and RgpF and an ABC transporter system embodied by RgpC and RgpD (2, 6), which were first characterized in Streptococcus mutans and are essential for synthesis of its cell surface-associated rhamnose-glucose polysaccharide (Rgp) (7, 8). In characterized ovococcal species, the variable region encodes the enzymatic machinery to synthesize the decorative side chain structure attached to the peptidoglycan-embedded rhamnan moiety (9–11). This genetic diversity gives rise to strain-level compositional and structural diversity in the associated rhamnose-containing CWPS (1, 8).

Streptococcus thermophilus is of substantial technological and economic importance due to its extensive application in both industrial and artisanal dairy fermentations (12, 13). In contrast to Lactococcus lactis, the rhamnose-containing CWPS structures of S. thermophilus, represented by Rgp, remain poorly characterized. Recent bioinformatic analysis of *rgp* loci present in sequenced S. thermophilus genomes has, however, revealed extensive diversity, which may be applied to distinguish between strains of the species (14, 15). These studies broadly classified S. thermophilus Rgp into one of five groups, designated A through E (14). However, this nomenclature was later revised following the development of a multiplex PCR system (16), which was designed to differentiate and classify S. thermophilus strains into one of four *rgp* genotypes, namely, Rgp1 through Rgp4 (where RGp1 is group B, RGp2 is group A, RGp3 is group D, RGp4 is group C and, by extension, RGp5 is group E) (14, 16).

Compositional analysis of the monosaccharides obtained from total cell wall fractions of the industrial strains STCH_12 and STCH_15 detected rhamnose (Rha), glucose (Glc), and galactose (Gal) in addition to *N*-acetylglucosamine (GlcNAc) and *N*-acetylmuramic acid (MurNAc) (17). Rgp chemical structures of just two S. thermophilus strains have been elucidated to date, i.e., those of St64987 (18) and UCCSt50 (19). In the current study, we determined the Rgp chemical structure of three additional S. thermophilus strains which harbor distinct *rgp* loci to establish the extent of structural diversity and to investigate if an *rgp* genotype to structure relationship may be established for this technologically important species.

## RESULTS

### Classification of S. thermophilus strains by Rgp multiplex PCR.

S. thermophilus has historically been regarded as a species of limited genetic diversity (20). However, recent reports suggested that while the core genome constitutes over 40% of the total gene content (21), there are regions of divergence, including the *rgp* biosynthetic cluster (14–17), the variable regions of which form the basis of the aforementioned genotyping multiplex PCR (16) that discerns four distinct genotypes.

In the present study, 70 S. thermophilus strains from the UCC collection were analyzed using this multiplex PCR system (16). Among these, 40, 38.57, 20, and 1.43% were assigned to Rgp1, -2, -4, and -3, respectively (Table 1). Representative strains of Rgp2 (strains UCCSt10 and UCCSt95), Rgp3 (strain UCCSt89), and Rgp4 (strain UCCSt12) were selected for whole-genome sequencing and comparative analysis of their associated *rgp* loci.

**TABLE 1:** Distribution of Rgp groups across the 70 UCC S. thermophilus strains as determined by Rgp multiplex PCR

Rgp genotype^*a*^	No. of strains	Distribution (%)
1	28	40
2	27	38.57
3	1	1.43
4	14	20

a The Rgp genotypes are based on the mPCR system described previously (16).

### Diversity among *rgp* loci present in Streptococcus thermophilus genomes.

**FIG 1:**
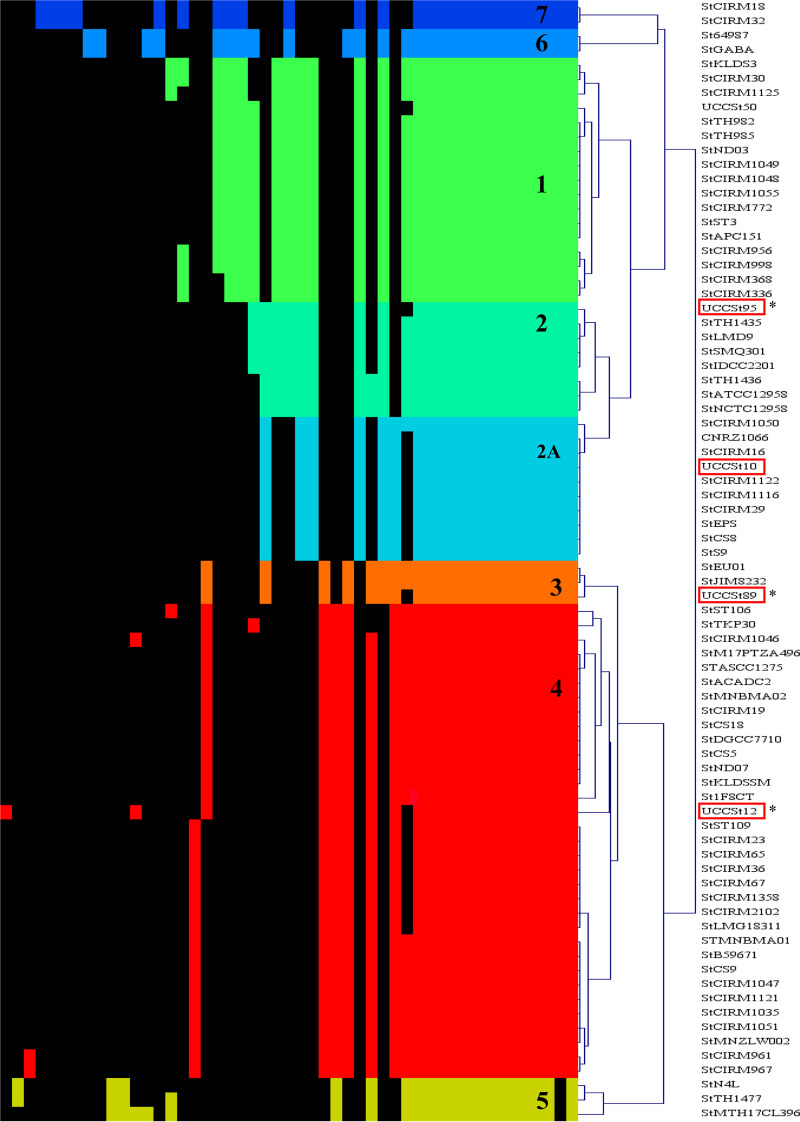
HCL analysis of the *rgp* loci of 78 S. thermophilus strains. The heatmap was generated on a protein family presence (color) or absence (black) basis. The assigned Rgp groups are indicated by color and an internal text marker. Representative strains from the UCC collection selected for genome sequencing are highlighted in red boxes. Representative strains from the UCC collection that were subjected to biochemical analysis as part of this study are indicated by an asterisk.

The *rgp* loci of 78 strains (74 from strains with publicly available complete genome sequences and 4 from strains whose genomes were sequenced in the context of the current study) were collated and compared using hierarchical clustering (HCL). This analysis revealed the presence of 49 distinct gene families within seven *rgp* genotypes (Fig. 1), representing five of the previously identified *rgp* genotypes (*rgp1* to -*5*) (14, 16) and two additional *rgp* genotypes (*rgp6* and *rgp7*). Overall, Rgp4 strains represented the majority, accounting for 42% of the analyzed strains. This outcome was in keeping with previous findings of Szymczak and colleagues, who reported Rgp4 strains as the most prevalent in an industrial strain collection (14). Furthermore, Rgp4 strains were also found to be dominant among those isolated from a range of fermented dairy products (22), representing 83.1% of all assessed S. thermophilus isolates.

**FIG 2:**
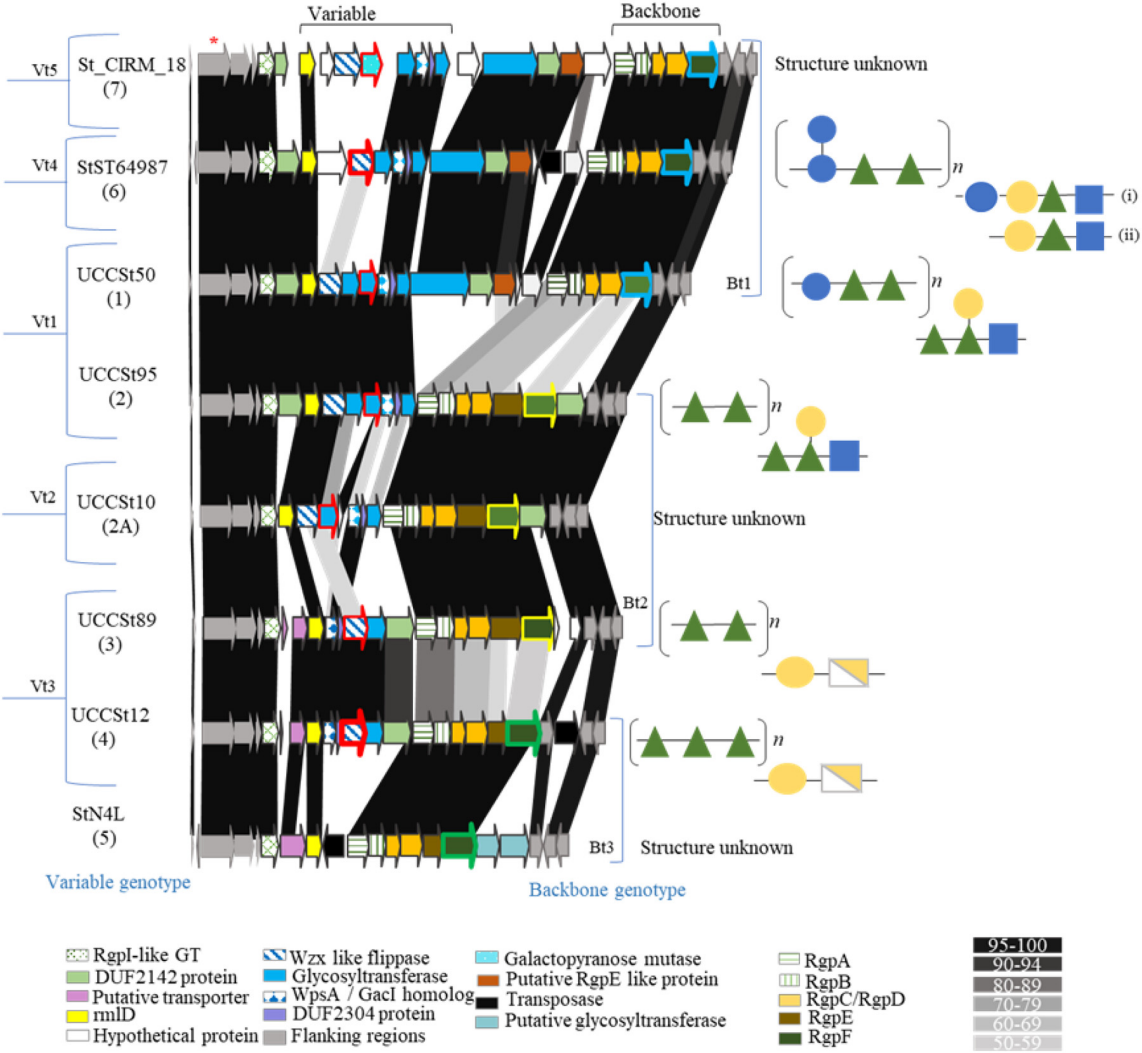
Schematic overview of the genomic organization and levels of genetic relatedness between *rgp* loci from S. thermophilus strains representative of Rgp1 through Rgp7. The predicted functions of each of the protein-encoding ORFs are color coded and indicated at the base of the figure. The *rgp* locus can be split into two distinct regions: the variable leftward (5′) end and the more conserved rightward (3′) end, which harbors genes relating to rhamnan backbone synthesis. The ORF from which the control primer for both multiplex PCR systems was designed is indicated by an asterisk. The unique ORFs used for variable-chain typing are outlined in red. The distinct ORFs used for *rgpF* genotyping are highlighted as follows; blue, *rgpF* genotype 1; yellow, *rgpF* genotype 2; green, *rgpF* genotype 3. Parentheses on the left indicate the variable-chain type as defined by multiplex PCR 1. Parentheses on the right indicate the *rgpF* genotype as defined by multiplex PCR 2. Schematic representations of the biochemical structures (when known) of both the polymerized rhamnan core and oligosaccharide are presented on the right. Monosaccharide symbols are based on those indicated by the Standard Nomenclature for Glycans (SNFG).

Among Rgp2 strains, there appears to be a subgroup (Rgp2A) (Fig. 1 and 2) which lacks genes that are predicted to encode a GT family 2 protein and a DUF2142 family protein. The Rgp6 and Rgp7 strains also harbor unique gene families within their associated variable regions. For example, the *rgp* loci of Rpg7 strains harbor a putative UDP-galactopyranose mutase-encoding gene within the 5′ variable region (Fig. 2). Remarkably, in Rgp5 strains, represented here by S. thermophilus N4L (Fig. 2), the variable 5′ region of the *rgp* locus is limited in size and lacks the glycosyltransferase-encoding gene content typically observed in the variable region of other *rgp* loci. However, the locus harbors two unique genes downstream of the rhamnosyltransferase-encoding gene, *rgpF*, that are predicted to encode polytopic membrane proteins, the first of which displays structural homology to known oligosaccharyltransferases (PDB 5OGl and 5EMZ_A). Based on their predicted functions, these gene products may play a role in Rgp biosynthesis or modification in Rgp5 strains.

### Architecture and gene content of the S. thermophilus *rgp* loci.

As previously reported by Hols et al., the *rgp* locus of S. thermophilus possesses a unique architecture, as genes predicted to encode the biosynthetic functions for the variable side chain structure appear to precede those associated with production of the rhamnan backbone (23). The latter genes encompass the rightward end of the bipartite S. thermophilus *rgp* locus and incorporate homologs of the well-characterized rhamnosyltransferase-encoding *rgpA*, *rgpB*, and *rgpF*. Furthermore, the rightward end of *rgp* loci of all strains assessed in this study harbored *rgpC* and *rgpD*, which together encode components of an ABC transport system (Fig. 2).

For strains belonging to Rgp2, -2A, -3, -4, and -5, homologs of *rgpE* were present and highly conserved at the intragroup level (Fig. 2). Recently, it was reported that S. thermophilus Rgp1 strains are characterized by the absence of an *rgpE* homolog at the same relative genomic position as that of Rgp2 through Rgp5 strains (Fig. 2) (15). The present study indicated that Rgp6 and Rgp7 strains also align with this architecture and share a high level of identity (≥90%) with Rgp1 strains across the 3′ end of the *rgp* locus (Fig. 2). Of note, the *rgp* loci of Rgp1, -6, and -7 strains harbor three unique genes in the central region which are predicted to encode a large, multidomain glycosyltransferase, a DUF2142 domain-containing polytopic membrane protein, and a glycosyltransferase which shares 45% identity (across 92% of the protein) with *rgpE* of Rgp2 strains (Fig. 2).

As RgpE is hypothesized to “cap” or regulate rhamnan chain length in L. lactis (24), the presence of a putative *rgpE* homolog suggests that these three genes may play a role in the biosynthesis of the polyrhamnose backbone. Despite an overall conserved architectural synteny, the region corresponding to rhamnan backbone biosynthesis of the assessed S. thermophilus *rgp* loci displayed a high level of intergroup disparity, and three distinct rhamnan backbone-associated genotypes were identified, based upon genetic identity (Fig. 2).

The leftward (5′) region of a given S. thermophilus *rgp* locus is predicted to encode functions associated with the biosynthesis of the variable side chain decoration that is attached to the rhamnan backbone polysaccharide. The first glycosyltransferase-encoding gene within the assessed S. thermophilus *rgp* loci is highly conserved between strains (Fig. 2) and shares >76% identity and 100% query coverage with *rgpI* of S. mutans, which is believed to be involved in the regulation of branching frequency of the glucose side chain decoration (25). A conserved homolog of *rmlD*, whose product is required for the final step in the dTDP-l-rhamnose biosynthetic pathway (26), is also located at the 5′ end (Fig. 2). Overall, the leftward ends of analyzed *rgp* loci displayed a high level of intergroup variation and diversity, particularly among glycosyltransferase-encoding genes. Interestingly, it was noted that where ≥95% homology existed between the 5′ region of distinct *rgp* loci, the 3′ region of such loci, which corresponds to rhamnan synthesis, nonetheless may have differed significantly (Fig. 2), indicating that these strains produce chemically distinct Rgp structures and prompting further investigation of the chemical diversity of the Rgp structures of dairy streptococcal strains.

### Elucidation of biochemical structures of selected S. thermophilus CWPS.

**FIG 3:**
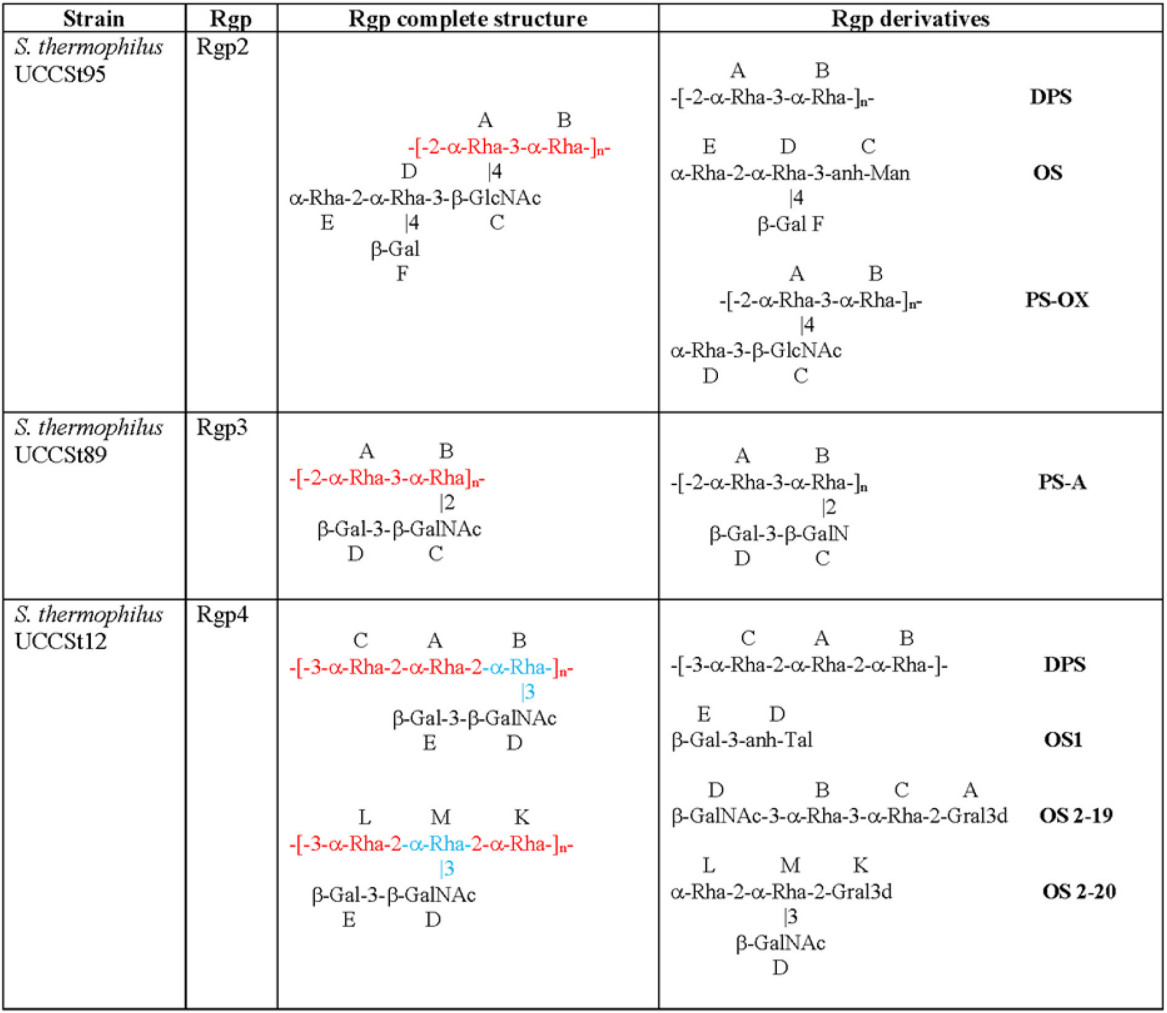
Chemical structures the CWPS rhamnan (Rgp) of S. thermophilus strains UCCSt95, UCCSt89, and UCCSt12. Red, rhamnan backbone polymer; blue, variable linkage position of the side chain structure.

Based on the presence of unique genetic content within the *rgp* loci, three strains (UCCSt95, UCCSt89, and UCCSt12, representing members of Rgp2, -3, and -4, respectively) were selected for biochemical analysis of their associated Rgp structures (Fig. 3; see also the supplemental material). The elucidated structures of the representative strains were subsequently compared to those of UCCSt50 and St64987 (18, 19), in order to establish a baseline for Rgp structural diversity. Strains representing Rgp5 or Rgp7 were unavailable for analysis in this study.

For S. thermophilus UCCSt95, the nuclear magnetic resonance (NMR) spectra of the Rgp preparation were heterogeneous. Methylation analysis showed the presence of a terminal, 2-linked, 3-linked, and 2,4-linked Rha, terminal Gal, and branched HexN as the major components, in addition to several minor components. The product was deacylated and deaminated, producing two components: a deaminated polysaccharide (DPS), corresponding to the rhamnan backbone composed of disaccharide repeating units (-2-α-Rha-3-α-Rha-), and OS, a branched tetrasaccharide with a 3-substituted 2,5-anhydro-mannose (anh-Man; product of the deamination of glucosamine) at the reducing end, corresponding to the rhamnan side chain (Fig. 3; see also Table S1 in the supplemental material). Smith degradation of the Rgp preparation produced a single polymeric product, PS-OX, which unambiguously established that the side chains were attached at position 4 of residue A (-2-α-Rha) of the rhamnan backbone (Fig. 3). Signals corresponding to side chains were also clearly visible in NMR spectra of intact CWPS, which allowed the assignment of its NMR spectra (Table S1) and the determination of the full structure of the repeating unit of the Rgp (Fig. 3).

**FIG 4:**
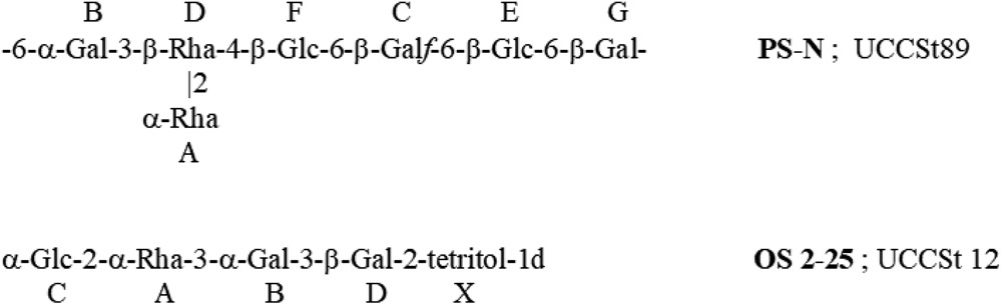
Chemical structures of EPS (PS-N) of S. thermophilus strain UCCSt89 and a product of Smith degradation of a putative EPS (OS 2-25) of S. thermophilus strain UCCSt12.

The S. thermophilus UCCSt89 preparation contained a mixture of two uncharged saccharidic structures and, based on NMR data, contained a single *N*-acetyl-amino sugar. The mixture was N-deacylated and separated on a cation exchange column to yield two pure polysaccharides, PS-N (neutral) and PS-A (amino), whose structures were fully elucidated by NMR. The amino-polysaccharide (PS-A) is a product of N-deacetylation of the branched rhamnan, having the (-2-α-Rha-3-α-Rha-) backbone identical to one of the Rgps of strain UCCSt95 and carrying disaccharide β-Gal-3-β-GalNAc branches at position 2 of the -3-α-Rha- residue B of the rhamnan backbone (Fig. 3; Table S2 and Fig. S1). The neutral PS-N component was shown to be composed of heptasaccharidic repeating units (Fig. 4; Table S3 and Fig. S1) and represented an additional polysaccharide, unrelated to Rgp.

The Rgp preparation of S. thermophilus UCCSt12 showed highly heterogeneous spectra, which were too complex to assign. Methylation analysis showed the presence of 2-linked, 3-linked, and 2,3-linked Rha, terminal Gal, and 3-linked GalNAc as the major components and a number of minor signals.

The Rgp was deacylated, desalted, and separated on Hitrap S cation exchange column. The charged (amino) fraction was deaminated and separated on a Sephadex G15 column, to give a DPS polymer and OS fraction, OS1, both of which were characterized by NMR (Fig. 3; Table S4). The anhTal at the reducing end of OS1 is the product of GalN deamination. These data indicated the presence of the rhamnan backbone, composed of a trisaccharide repeating unit (-3-α-Rha-2-α-Rha-2-α-Rha-), and disaccharide side chains, β-Gal-3-β-GalNAc. A small amount of a rhamnan identical to DPS was isolated as a neutral fraction on a cation exchange column, indicating the presence of a linear nonbranched rhamnan in the Rgp preparation.

In order to identify the site of attachment of the side chains to the rhamnan backbone, the Rgp preparation was subjected to Smith degradation, and the products were separated on Sephadex G15. Two fractions, fr. 1 and fr. 2, were collected. Both were complex mixtures of oligosaccharides, indicating that the original Rgp-derived rhamnan polymer main chain was degraded by periodate oxidation. They were separated by HILIC chromatography. Several pure oligosaccharides were obtained from fr. 2, among which the major components were tetrasaccharides OS 2-19 and OS2-20 with glyceraldehyde (2-Gral3d), a product of Smith degradation of a 2-substituted sugar, at the reducing end (Fig. 3). Their NMR spectra were completely assigned (Table S4). All these data combined indicated that the original structure was a mixture of rhamnan repeating units, with differently positioned branches (Fig. 3). Longer oligosaccharides that were also isolated after oxidation indicated that branches can be attached at either of the 2-α-Rha moieties in the rhamnan backbone (Fig. 3).

Oxidation of the UCCSt12 Rgp preparation also produced a significant amount of an oligosaccharide, OS2-25 (Fig. 4; Table S5) that was unrelated to the above-described Rgp polymer. It was possibly a product of Smith degradation of another polysaccharide (putative EPS) of strain UCCSt12. Its signals were well visible in the spectra of the original Rgp preparation.

To summarize, structural studies of Rgp preparations of strains UCCSt95, UCCSt89, and UCCSt12, representing Rgp2, -3, and -4, showed presence of Rgp polymers with a similar molecular architecture. Rgp are composed of a di- or trisaccharide repeating unit rhamnan backbone that carries side chains with an amino sugar at the branching point. Unlike the Rgp of previously characterized strains St64987 (18) and UCCSt50 (19), the backbone did not contain Glc. The side chains represented a branched tetrasaccharide for the Rgp of UCCSt95 (Rgp2) and a disaccharide, β-Gal-3-β-GalNAc-, for the Rgp of strain UCCSt89 or UCCSt12 (Rgp3 and -4). The attachment of the side chains to the rhamnan backbone was variable.

Interestingly, in strains UCCSt12 and UCCSt89, in addition to branched Rgp components, additional novel neutral polysaccharides were identified (Fig. 4). Thus, we determined that S. thermophilus strain UCCSt89 and UCCSt12 cell walls contain two distinct polysaccharide components, Rgp and EPS, a finding which has previously also been described for St64987 (18).

### Distinct *rgp* genotypes are linked to unique biochemical Rgp structures in S. thermophilus.

The *rgp* loci of S. thermophilus UCCSt95 (Rgp2) and UCCSt50 (Rgp1) are almost identical across the variable (5′) side chain-associated region yet differ significantly across the 3′ rhamnan backbone-associated region (Fig. 2). Consistent with this, the structural data confirmed that UCCSt95 and UCCSt50 possess identical tetrasaccharide side chain structures yet distinct rhamnan core components (Fig. 2). In keeping with *rgp* locus analysis, the rhamnan of UCCSt95 (Rgp2) is a repeating -2-α-Rha-3-α-Rha- disaccharide (Fig. 2 and 3) and is identical to that of UCCSt89 (Rgp3); however, the decorative side chain structure of the latter is a disaccharide unit of β-Gal-3-β-GalNAc (Fig. 2 and 3).

The structure of Rgp4 strain UCCSt12 is a repeating trisaccharidic -3-α-Rha-2-α-Rha-2-α-Rha rhamnan polymer and a disaccharidic side chain decoration composed of β-Gal-3-β-GalNAc, which may be attached at one of two identified branch positions (Fig. 3). Of note, the structure of the side chain decoration was identical to that of Rgp3 strain UCCSt89, an outcome which was consistent with the high degree of genetic relatedness observed across the 5′ region of *rgp* loci of these strains (Fig. 2 and 3).

It has recently been shown that the *cwps* genotype of L. lactis strains is linked to the biochemical structure of the polysaccharide, based on the number of encoded glycosyltransferases, the identification of a conserved priming glycosyltransferase, and the presence or absence of unique identifiers and functions (9). For example, the presence of an encoded UDP-galactopyranose mutase correlates to the presence of a Gal*f* residue in the biochemical structure of the variable side chain present in the CWPS of several L. lactis C-type strains (9). The bioinformatic and structural data obtained for the S. thermophilus strains analyzed in this study alluded to a similar genotype-to-structure relationship for this species. This hypothesis is supported by the following: (i) an identical biochemical structure has been elucidated for strains which share ≥95% identity across the variable side chain- or rhamnan-specifying regions, (ii) the number of encoded glycosyltransferases within the variable region can be directly correlated with the monosaccharide composition of the decorative side chain, and (iii) all strains assessed harbored homologs of the L. lactis MG1363 side chain initiating glycosyltransferase, encoded by *wpsA* (24), which we previously functionally characterized for the Rgp1 strain UCCSt50 (19). In L. lactis, WpsA is responsible for the transfer of a GlcNAc moiety to the lipid carrier undecaprenyl-phosphate (Und-P), producing Und-P-GlcNAc, which serves as the foundation on which the side chain structure is cytosolically assembled (24). The WpsA homologs of S. thermophilus Rgp1, -2, -6, and -7 representative strains share 52% identity with that of L. lactis WpsA; however, the level of identity was reduced to 37% for those encoded by Rgp2A, -3, and -4 strains. Notably, the WpsA homologs encoded by Rgp2A, -3, and -4 strains also shared a reduced level of identity (45%) when compared to that of Rgp1, -2, -6, and -7 (Fig. 2). As such, it was hypothesized that the reduced level of identity reflects the distinct nature of the monosaccharide that is transferred to the lipid carrier at the initiating stage of side chain biosynthesis. Consistent with this is the presence of a GlcNAc moiety at the branching point of the structure in Rgp1, -2, and -6 strains (and indeed that of L. lactis MG1363) (9, 19, 24), while strains belonging to Rgp3 and -4 possess a GalNAc moiety at their respective branch points.

### Proposed biosynthetic pathway for Rgp synthesis in Streptococcus thermophilus.

**FIG 5:**
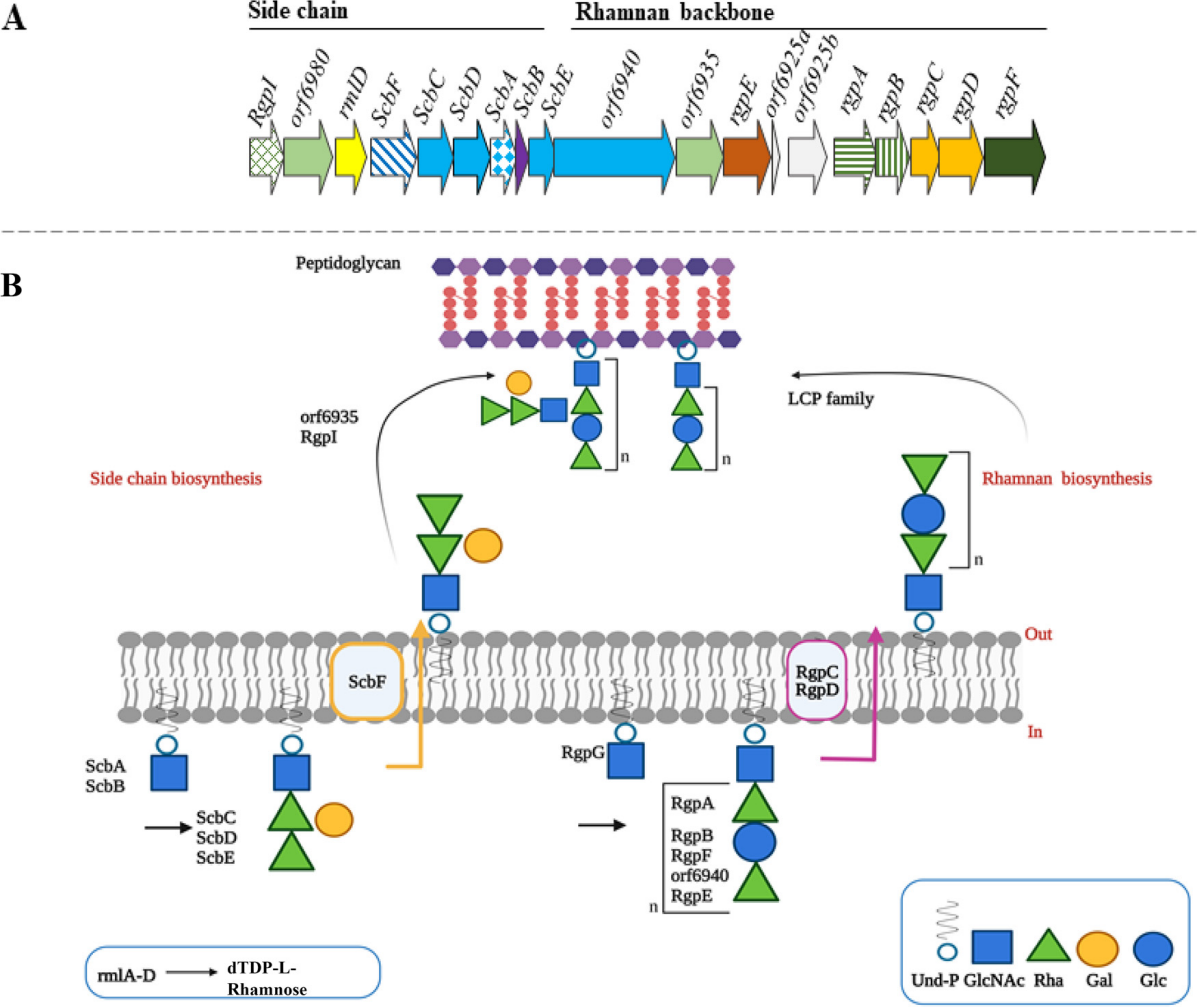
(A) Schematic representation of the *rgp* locus of S. thermophilus UCCSt50. Predicted functions of the encoded proteins are indicated as follows: green hatch, RgpI; light green, DUF2142 membrane protein; yellow, tDTP-l-Rha synthesis; blue stripe, *wzx*-like flippase; blue, glycosyltransferase (GT2); blue diamond, WpsA homolog; purple, WpsB homolog; navy, GT; orange, putative RgpE; green stripe, RgpA and RgpB; gold, RgpC and RgpD transporter system; dark green, RgpF; white, uncharacterized or interrupted. (B) Schematic overview of the proposed biosynthesis pathway in the S. thermophilus Rgp1 representative strain UCCSt50. A detailed description of each stage of the proposed pathway is provided in the main text. Images was created using BioRender.

Recently, detailed biosynthetic pathways of rhamnose-containing CWPS of L. lactis MG1363, L. lactis IL-1403, Enterococcus faecalis V583, and Streptococcus pyogenes have been proposed (5, 9, 24, 27). Functional genome analysis of the *rgp* loci of representative strains coupled with the elucidated biochemical structures facilitates an analogous assembly pathway to be put forward for the Rgp of S. thermophilus (Fig. 5; Table S6). Similar to L. lactis MG1363 (9, 24), we propose that the complete Rgp structure of S. thermophilus is assembled from two distinct but converging pathways—that of the rhamnan backbone and that of the side chain decoration, which are further discussed below.

### The polyrhamnose backbone.

Th proposed polyrhamnose backbone biosynthetic route relies on an intracellular pool of the nucleotide precursor sugar dTDP-l-rhamnose, whose biosynthesis is performed by the products of the *rml* operon (26–31). While *rmlD* is located within the *rgp* loci of all S. thermophilus Rgp representative strains (Fig. 2), *rmlA* to -*C* are located outside of the *rgp* locus. Additionally, the initiation of rhamnan synthesis is dependent on a TagO-like protein to catalyze the transfer of a GlcNAc moiety from UDP-GlcNAc to Und-P to form the precursor Und-P-P-GlcNAc. In S. mutans this function is performed by RgpG (32), and a TagO/RgpG-encoding gene (*stu163*) has been identified in S. thermophilus LMG18311 (33). In the present study, a *stu163* homolog was present in the genome of UCCSt50 (*orf01050*), suggesting that S. thermophilus UCCSt50 also initiates rhamnan backbone synthesis through the activity of an encoded RgpG homolog.

Subsequent to RgpG-mediated initiation, RgpA is believed to transfer the first rhamnose residue to the Und-P-P-GlcNAc foundation (Fig. 5). The remaining glycosyltransferases, RgpB and RgpF, polymerize the rhamnan backbone through the iterative addition of individual monosaccharide units. In L. lactis MG1363, RgpE is believed to prevent further elongation of the rhamnan chain by capping the UDP-linked polymer at the nonreducing end, thus regulating rhamnan chain length (24). Putative homologs of RgpE are present in the rhamnan backbone region of all S. thermophilus strains assessed in this study (Fig. 2), and it may therefore be hypothesized that rhamnan chain length is also regulated by RgpE in S. thermophilus.

Following intracellular synthesis, the rhamnan backbone polymer is transported across the membrane via the RgpC/D-dependent ABC-type transport system (Fig. 5). In the case of L. lactis and indeed E. faecalis V583 (2, 5, 24), LCP family enzymes are believed to be responsible for the incorporation of the rhamnan backbone unit into the peptidoglycan layer. Given the parallels in both genomic architecture and rhamnan biosynthesis in these species, it is likely that a S. thermophilus-encoded LCP family protein also performs this function.

### The variable side chain.

Biosynthesis of the decorative side chain structure in UCCSt50 is believed to be initiated by the side chain biosynthesis (Scb) protein A, ScbA_UCCSt50_, and its associated activator, ScbB_UCCSt50_ (Fig. 2). As detailed previously (19), these proteins initiate side chain synthesis through the addition of a GlcNAc (or GalNAc in the case of UCCSt12 and UCCSt89 [Fig. 2 and 3]) moiety to the lipid carrier Und-P, representing functional homologs of L. lactis WpsA/B and S. pyogenes GacI/J (24, 27). The subsequent elongation of the UCCSt50 side chain is believed to be a function of the glycosyltransferases encoded by *scbC*_UCCSt50_, *scbD*_UCCSt50_, and *scbE*_UCCSt50_ (where scb refers to side chain biosynthesis), which are predicted to sequentially add an individual monosaccharide to the lipid-linked GlcNAc foundation (Fig. 5; Table S6).

Although it is not possible to confirm the exact nature of the substrate for each of the glycosyltransferases encoded by *scbC*_UCCSt50_, *scbD*_UCCSt50_, and *scbE*_UCCSt50_, and thus the order of assembly, it remains apparent that their activity, coupled with that of the initiating glycosyltransferase ScbA_UCCSt50_, will produce an undecaprenyl (Und-P)-linked tetrasaccharide subunit. This lipoglycan precursor, following membrane translocation, is presumed to serve as the substrate to attach the tetrasaccharide to the rhamnan backbone, thus forming the experimentally determined side chain of the UCCSt50 Rgp (Fig. 5). Translocation of Und-P-linked saccharidic side chain precursors across the cytoplasmic membrane is typically performed by an integral membrane protein which shares sequence or structural similarity to the Wzx-like flippase of Escherichia coli (34), being involved in the export or “flipping” of such Und-P-linked glycan subunits to the outer membrane during O-antigen synthesis. ScbF, which contains 12 predicted transmembrane helices, shares significant structural similarity to the Escherichia coli lipid II flippase MurJ (PDB 6CC4). Similar bioinformatic and topological outputs have been reported for the L. lactis MG1363-encoded flippase, WpsG (24). It is, therefore, proposed that *scbF*_UCCSt50_ encodes a Wzx-like flippase which translocates the Und-P-linked tetrasaccharide subunit across the cytoplasmic membrane (Fig. 5; Table S6). Although a putative Wzx-like flippase-encoding gene was present in each representative *rgp* locus, with the exception of the Rgp5 strain StN4L, in which most of the variable region appeared to be absent (Fig. 2), a high degree of intergroup sequence divergence was apparent (Fig. 2), which may have been indicative of functional specificity based on the composition of the decorative side chain structure.

While certain CWPS-associated decorative structures of L. lactis (predominantly those belonging to CWPS type C) and those of the enterococcal antigen polysaccharide are known to be polymerized (5, 9, 24), the characterized glycan decorations of the S. thermophilus rhamnan backbone are not, a finding which was supported by the absence of a Wzy-like polymerase-encoding gene within the *rgp* locus of UCCSt50 (Fig. 2; Table S6).

The final stage in Rgp assembly is the regulated attachment of the decorative side chain to the rhamnan backbone, thus producing a mature Rgp structure. The *rgp* locus of S. thermophilus UCCSt50 harbors two genes, *orf6980*_UCCSt50_ and *orf6935*_UCCSt50_, whose products share topological properties with that of WpsJ, the polytopic GT-C fold glycosyltransferase of L. lactis MG1363 which is believed to be responsible for the transfer of the polymerized decorative chain to the rhamnan backbone (24). Because *orf6935*_UCCSt50_, whose product harbors nine transmembrane helices, is located within the rhamnan-specifying region of the UCCSt50 *rgp* locus, we propose that its product completes the biosynthesis of Rgp1 via the attachment of the tetrasaccharide decoration to the rhamnan backbone. Regulation of the decorative side chain branching frequency on the rhamnan backbone is most likely a function of the encoded RgpI, as it resembles the enzyme known to regulate glucose branching frequency of the rhamnan backbone in S. mutans with 76% sequence identity (25). Three putative genes, *orf6925a*_UCCSt50_, *orf6925b*_UCCSt50_ (manually annotated at positions 1332284 to 1331268), and *orf6980*_UCCSt50_, remain functionally unassigned, and further studies are required to confirm their role, if any, in Rgp biosynthesis.

### Development of a two-step multiplex PCR method to differentiate variable side chain grouping and rhamnan backbone genotypes.

**FIG 6:**
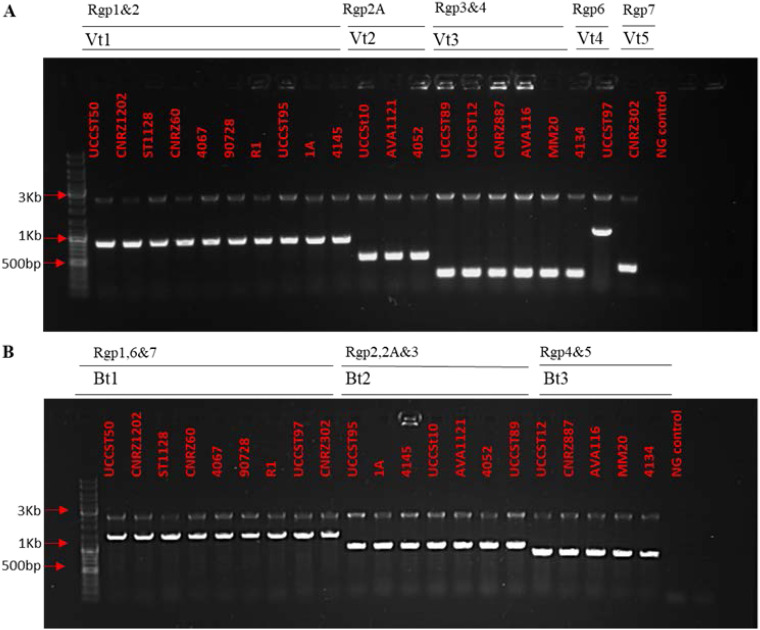
(A) Application of multiplex PCR systems 1 and 2 across 21 S. thermophilus strains for classification based on the variable side chain-encoding region. Lane 1, molecular weight marker; lanes 2 to 11, variable chain genotype 1; lanes 12 to 14, variable chain genotype 2; lanes 15 to 20, variable chain genotype 3; lane 21, variable chain genotype 4; lane 22, variable chain genotype 5; lane 23, negative control. The variable chain types (Vt) and the overall associated Rgp to which they align are indicated in the headings (lower and upper, respectively). Individual S. thermophilus strain names are indicated in red text. (B) Classification based on *rgpF* backbone genotype (Bt). Lane 1, molecular weight marker; lanes 2 to 10, Bt1; lanes 11 to 17, Bt2; lanes 18 to 22, Bt3; lane 23, negative control. The *rgpF* genotypes and the associated Rgp to which they aligned are indicated in the headings (lower and upper, respectively). Individual S. thermophilus strain names are indicated in red.

The HCL analysis and comparative genomic assessment of the *rgp* loci of representative S. thermophilus strains performed in this study confirmed a significant level of diversity between the defined *rgp* genotypes, not only across the 5′ variable side chain-encoding region but also across the 3′ region, which is predicted to encode the biosynthetic functions of the rhamnan backbone (Fig. 2). Furthermore, a “mix and match” of the rhamnan-encoding and decorative side chain-encoding regions of the bipartite *rgp* cluster was evident (Fig. 2). The previously described Rgp multiplex PCR system does not have the capacity to distinguish between strains with different combinations of rhamnan-encoding and decorative side chain-encoding regions, which would allow for informed predictions of overall Rgp composition and structure. To capture such levels of (anticipated) genotypic diversity, a two-step multiplex PCR system was developed. First, unique regions within the variable side chain region of each *rgp* genotype (five variable genotypes identified) (Fig. 2) were used to design genotype-specific primers for multiplex PCR 1. These primers were applied to classify the variable side chain genotype of 21 S. thermophilus strains from the UCC strain collection (Table S7), among which 10 (47.6%), 3 (14.3%), 6 (28.6%), 1 (4.8%), and 1 (4.8%) strains were classified as possessing variable genotype 1 to 5, respectively (Fig. 6A and Table 2). Notably, strains UCCSt97 and CNRZ302, which had been formerly classified as Rgp1 strains (Table 1) (16), were found to belong to the newly assigned Rgp6 and Rgp7, respectively, based on amplicon size and distinct gene content (Fig. 6A). Such results highlighted the improved discriminatory power of the newly designed Rgp primer sets.

**TABLE 2:** Summary of variable and backbone genotypes assigned to 21 S. thermophilus strains via multiplex PCRs 1 and 2 and the proposed binomial naming system for strain classification

Strain	Variable type	Backbone type	Proposed name	Rgp group^*a*^
S. thermophilus UCCSt50	Vt1	Bt1	V1B1	1
S. thermophilus CNRZ1202	Vt1	Bt1	V1B1	1
S. thermophilus ST128	Vt1	Bt1	V1B1	1
S. thermophilus CNRZ760	Vt1	Bt1	V1B1	1
S. thermophilus 4067	Vt1	Bt1	V1B1	1
S. thermophilus 90728	Vt1	Bt1	V1B1	1
S. thermophilus R1	Vt1	Bt1	V1B1	1
S. thermophilus UCCSt95	Vt1	Bt2	V1B2	2
S. thermophilus 1A	Vt1	Bt2	V1B2	2
S. thermophilus 4145	Vt1	Bt2	V1B2	2
S. thermophilus UCCSt10	Vt2	Bt2	V2B2	2A
S. thermophilus AVA1121	Vt2	Bt2	V2B2	2A
S. thermophilus 4052	Vt2	Bt2	V2B2	2A
S. thermophilus UCCSt89	Vt3	Bt2	V3B2	3
S. thermophilus UCCSt12	Vt3	Bt3	V3B3	4
S. thermophilus CNRZ887	Vt3	Bt3	V3B3	4
S. thermophilus AVA116	Vt3	Bt3	V3B3	4
S. thermophilus MM20	Vt3	Bt3	V3B3	4
S. thermophilus 4134	Vt3	Bt3	V3B3	4
S. thermophilus UCCSt97	Vt4	Bt1	V4B1	6
S. thermophilus CNRZ302	Vt5	Bt1	V5B1	7

a The Rgp grouping is based on the mPCR system described previously (16).

Multiplex PCR 2 was developed based on a previously reported observation that *rgpF* is a divergent gene within the rhamnan backbone-specifying region of S. thermophilus *rgp* loci (15). Three rhamnan-encoding regions were identified in this study (Fig. 2 and 6B), and these correlated with genotype-specific *rpgF* genes (*rgpF* genotype 1, 2, or 3). Of the evaluated strains, 42.8% possessed *rgpF* genotype 1, while 33.3% and 23.8% of the strains possessed *rgpF* genotypes 2 and 3, respectively (Table 2).

While the previously reported Rgp multiplex PCR system (16) provided a broad strain classification based on overall *rgp* genotype, it could not differentiate between Rgp1, -6, and -7 strains, nor did it distinguish between strains belonging to Rgp2 or its associated subgroup, 2A. Furthermore, it did not allow unique combinations of rhamnan-associated and side chain-associated gene clusters that are anticipated though still to be identified. For example, gene cluster combinations of variable type 1 and *rgpF* backbone type 3 (V1B3, V2B1, V2B3, V3B1, V4B2, V4B3, V5B2, and V5B3) were not detected within the remit of this study (Table 2). Therefore, the development of this two-step multiplex PCR system facilitated a detailed characterization of strain collections based on the two functionally distinct regions of the *rgp* locus from different strains. In addition, loss of the variable side chain structure has recently been associated with a phage resistance phenotype (18, 19), and as such, the application of multiplex PCR 1 for the classification of strains based upon their associated variable genotype will aid in predicting phage-host interactions and subsequent strain selection from the industrial perspective.

## DISCUSSION

In this study, we assessed the diversity of the *rgp* loci of 78 S. thermophilus strains via HCL analysis. Through this approach, two novel *rgp* genotypes (designated here as Rgp6 and Rgp7) beyond the five previously described (14, 16) were identified. The novel *rgp* loci possessed distinct gene content within the variable side chain-encoding regions (Fig. 1 and 2). As has now become evident, the S. thermophilus *rgp* locus is a bipartite genetic entity, being composed of two functionally distinct parts which appear to be capable of mix and match arrangements, and we therefore propose that a binomial naming system be introduced which represents both elements, the variable (V) and backbone (B) (Table 2). Although the overall distribution of Rgp5, Rgp6, and Rgp7 strains is currently low, their presence indicates that additional genetic variation among S. thermophilus *rgp* loci exists and may expand further as additional genome data emerge. The level of intergroup diversity observed for the S. thermophilus *rgp* loci is reminiscent of that of the L. lactis *cwps* gene clusters. Primarily owing to their established role as phage receptors for multiple lactococcal phage species, including the prolific 936 phages, *cwps* loci of L. lactis have been subjected to intense genomic scrutiny and biochemical structural analyses (2, 3, 9, 10, 24), which have revealed a genotype-to-structure relationship. The biochemical analysis of the Rgp produced by Rgp2, -3, and -4 representative strains undertaken as part of this study, in addition to those previously elucidated for Rgp1 (19) and Rgp6 (18), suggests that S. thermophilus *rgp* loci also adhere to a genotype-to-structure association model (Fig. 2 and 3).

The decorative side chain structures vary significantly, ranging from complex tetrasaccharide structures (Rgp1, Rgp2, and Rgp7) to simpler disaccharidic decorations akin to those of pathogenic streptococci (Fig. 2 and 3) (6, 27, 35). Similarly, the rhamnan backbone component of the S. thermophilus Rgp structures was shown to display an unexpected variability in terms of monosaccharide composition and modification (Fig. 2 and 3). For example, the region encoding the rhamnan component of Rgp1 and Rgp7 strains shared a high level of architectural synteny and sequence similarity, yet the structure of the Rgp7 rhamnan possessed a glucose modification. The similar glucose modification on the rhamnan backbone of certain L. lactis strains has been reported to be a function of a three-component glycosylation system (TGS) which incorporates the glycosyltransferases CsdA and CsdB and a flippase, CflA (9, 36). As such, it is possible that the glucose modification of the Rgp7 rhamnan backbone is mediated by genomic elements located outside of the *rgp* locus. Extensive levels of CWPS/Rgp diversity among ovococcoid bacteria are often attributed to adaptation mediated by external selective pressures, in particular phage predation (9), and although the S. thermophilus-encoded CRISPR-Cas systems provide a highly adaptable phage defense for the species (37), recent evidence has confirmed that nonsense mutations in Rgp and EPS-associated encoded glycosyltransferases (14, 18, 19) are a key response to phage-imposed selective pressure for the species. It could therefore be hypothesized that *rgp* loci of S. thermophilus have also acquired such diversity as a response to external environmental pressures, in particular that caused by phage attack. The recent establishment of detailed biosynthesis pathways for rhamnose-containing polysaccharides of enterococci, lactococci, and pathogenic streptococci (5, 24, 27) has revealed a common biosynthetic mechanism among these species. Through a bioinformatic survey of the Rgp-associated genes from the Rgp1 strain, UCCSt50, it was evident that this species encodes similar biosynthetic components. Based on these findings, we proposed a biosynthetic model for the complete Rgp structure in S. thermophilus (Fig. 5). The presence of conserved elements, including homologs of the priming glycosyltransferase WpsA, a Wzx-like flippase, and variable glycosyltransferases, may allow this model to be adapted to all known Rgp groups. Finally, we used the genomic and biochemical data acquired during this study to refine the current Rgp multiplex system (16) to allow for a detailed, dual classification of S. thermophilus strains. As the *rgp* locus of S. thermophilus has been directly implicated in the initiating stages of phage infection for at least two brussowviruses (14, 19), the ability to classify strains on this basis is of particular relevance in an industrial context and will enable the development of rational, rotating starter systems specifically designed to limit phage proliferation and fermentation failure.

### Conclusions.

The current study has revealed an unexpected level of structural diversity and complexity in the rhamnose-containing CWPS (Rgp) of S. thermophilus. This diversity is reflected in the genomic content of the associated *rgp* loci, with particular reference to the number of encoded glycosyltransferases and the saccharidic composition of the associated biochemical structure(s). Extensive genetic diversity has also been observed across S. thermophilus *EPS* loci in several studies. Therefore, it is now evident that the genomic loci associated with the biosynthesis of S. thermophilus cell wall-associated polysaccharides are dynamic, highly complex, and underpin the chemical and functional diversity of these structures.

In line with the current dogma of the genome-structure relationship which has been established for *cwps* loci of L. lactis (9), it is now possible to make predictions relating to the streptococcal Rgp structures, including oligosaccharide chain length, monosaccharide composition, and the presence of unique identifiers such as Gal*f.* Future functional studies are required to experimentally confirm these findings, and structural biochemical analysis of Rgp2A, Rgp5, and Rgp7 representative strains is required to complete the structural data set for all known S. thermophilus Rgp groups. Furthermore, continual updates to the newly developed mPCR systems will further extend their discriminatory power. As additional genome sequences become available, it is envisaged that the diversity of S. thermophilus *rgp* loci will further increase, revealing the true extent of the genomic and structural flexibility pertaining to rhamnose-containing polysaccharides in this important dairy species.

## MATERIALS AND METHODS

### Bacterial growth conditions.

S. thermophilus strains UCCSt12, UCCSt89, and UCCSt95 were grown overnight in M17 broth (Oxoid, United Kingdom) supplemented with 0.5% lactose at 42°C. Culture volumes ranged from 4 to 16 liters (strain dependent), and cells were harvested by centrifugation at 4,424 × *g* at 4°C for 30 min. The resulting cell pellets were washed in 200 mL ice-cold sterile, distilled water (dsh_2_O) with a subsequent and final wash in 50 mL ice-cold dsH_2_O.

### Preparation and structural analysis of CWPS isolated from S. thermophilus strains.

All S. thermophilus strains were preextracted with cold trichloroacetic acid and then extracted with hot 0.01 M and 0.1 M HCl, as described previously (18). Most abundant 0.01 M HCl extracts were fractionated on a Sephadex G-50 column (1.6 by 90 cm) to give the total CWPS preparations. For strain St12, optimal yields were obtained by extraction of freeze-dried cells (0.5 g) with 48% hydrofluoric acid (HF) (3.5 mL, 5°C, 48 h with stirring). The suspension was diluted with water and centrifuged, and the supernatant was dialyzed, freeze-dried, and purified on a Sephadex G-50 column.

Monosaccharide and methylation analyses were performed as described for S. thermophilus St64987 (18). For Smith degradation, CWPS preparations of UCCSt95 and UCCSt12 were oxidized with sodium periodate (NaIO_4_, 0.1 M 24 h, 25°C), reduced with sodium borodeuteride (NaBD_4_), hydrolyzed with acetic acid (AcOH; 2%, 1.5 h, 100°C), and isolated on a Sephadex G-15 column. For strain UCCSt95, only polymer PS-OX was obtained. For UCCSt12, two fractions, 1 and 2, were collected. Both were complex mixtures of oligosaccharides. They were separated by HILIC chromatography on a Tosoh amide-80 column in a 70-to-40% MeCN gradient. Several pure oligosaccharides was obtained from fr. 2, but fr. 1 gave no clean product.

**Gel chromatography.** Gel chromatography was performed on a Sephadex G-50 column (1 by 40 cm or 1.6 by 80 cm) in 0.1% acetic acid, a Sephadex G-15 column (1.6 by 60 cm), or a Biogel P10 column (2.5 by 60 cm) in 1% acetic acid and monitored with a refractive index detector (Gilson).

**Anion-exchange chromatography.** Polysaccharide sample (up to 50 mg) was injected into HiTrap Q column (Amersham; two columns by 5 mL each connected together) in water at 3 mL/min, washed with water for 5 min, then eluted with a linear gradient from water to 1 M NaCl over 1 h with UV detection at 220 nm. We performed a spot test on a silica thin-layer chromatography plate with development by dipping in 5% H_2_SO_4_ in ethanol and heating with a heat gun until brown spots became visible. Samples were desalted on a Sephadex G-15 column (1.6 by 60 cm) in 1% AcOH with a refractive index detector.

**HILIC chromatography.** Samples were dissolved in water (20 μL), diluted with MeCN to 80% MeCN, and injected into a Tosoh Amide-80 column (4.6 by 150 mm; MeCN in a water gradient from 80% to 40% over 40 min at 1 mL/min, UV detector at 220 nm). Each tube by 1 min was tested for the presence of carbohydrates by spot test as described above.

**NMR spectroscopy.** NMR experiments were carried out on a Bruker Avance III 600 MHz (^1^H) spectrometer with a 5-mm Z-gradient probe with acetone internal reference (2.225 ppm for ^1^H and 31.45 ppm for ^13^C) using standard pulse sequences cosygpprqf (gCOSY), mlevphpr (TOCSY, mixing time 120 ms), roesyphpr (ROESY, mixing time 500 ms), hsqcedetgp (HSQC), hsqcetgpml (HSQC-TOCSY, 80 ms TOCSY delay), and hmbcgplpndqf (HMBC, 100-ms-long range transfer delay). Resolution was kept <3 Hz/pt in F2 in proton-proton correlations and <5 Hz/pt in F2 of H-C correlations. The spectra were processed and analyzed using the Bruker Topspin 2.1 program.

Monosaccharides were identified by COSY, TOCSY, and NOESY cross-peak patterns and ^13^C NMR chemical shifts. Amino group location was concluded from the high-field signal position of aminated carbons (CH at 45 to 60 ppm). Connections between monosaccharides were determined from transglycosidic NOE and HMBC correlations.

**Alkaline deacylation.** Polysaccharide samples in polypropylene vials were dissolved in 4 M KOH (4 mL each), kept overnight at 120°C, and neutralized with 2 M HCl. Precipitated material was removed by centrifugation, and deacylated material was isolated by gel chromatography on a Biogel P10 column (2.5 by 60 cm).

**Deamination.** Deacylated PS was deaminated with NaNO_2_-AcOH; to the solution of PS in water (2 mL), NaNO_2_ (20 mg) and acetic acid (0.1 mL) were added, mixtures were stirred to dissolve components, and after 1 h at room temperature products were isolated by gel chromatography on a Sephadex G-15 column, yielding the deaminated polysaccharide (DPS) and a mixture of oligosaccharides.

### DNA preparation, sequencing, assembly, and annotation.

DNA preparation and genome assembly methods employed for S. thermophilus strains UCCSt10, UCCSt12, and UCCSt95 were the same as those reported for strain UCCSt50 (19), while genome sequencing of UCCSt89 was performed using an Illumina MiSeq platform. Detailed annotation of the *rgp* loci of the above-mentioned strains was performed using a combination of BLASTP, Pfam, and HHpred, and transmembrane helices were detected using the TMHMM server V.2.0 (38–41).

### Classification of S. thermophilus strains using *rgp* locus-specific multiplex PCR.

Seventy S. thermophilus strains from the UCC collection were classified into one of four *rgp* genotypes based on a previously described multiplex PCR system (16), using the following conditions: 95°C for 10 min, followed by 35 cycles of 95°C for 15 s, 55.0°C for 30 s, and 72°C for 1 min, followed by a final extension step at 72°C for 10 min, using the Rgp classification primer set listed in Table 3.

**TABLE 3:** Primers for Rgp classification of S. thermophilus strains

Primer	Target *rgp*	Sequence (5′–3′)	Amplicon (bp)	Reference
*rgp* classification				
RGPposF	All	CAGGTGCAAATGGCCAACTCG		16
RGPposR		CTTGCCATGTTGGGATGAC	801	16
RGPgroup1F	1	GGATGATGGTTCGACGGATAG		16
RGPgroup1R		CCGCTCTTCCAAAACCATGA	631	16
RGPgroup2F	2	GTGAAGAGTCAGAAGACGAAT		16
RGPgroup2R		CAAAGGCCCCGATGGTATT	464	16
RGPgroup3F	3	GAGGAAGCAACAGATAAACGA		16
RGPgroup3R		GACCAATTGGTCCACAAAAGT	303	16
RGPgroup4F	4	CTCCTCGTACTCACCCAC		16
RGPgroup4R		GCACAAGATACAGCTCGTTAC	162	16
Both multiplex systems				
MSControl F	All	GCTGGTCGTAATTACCTCG		This study
MSControl R	All	CAACATCTTCCAAGGTACG	2,724	This study
Multiplex system 1				
Var1F	1, 2	GTATATAATGCACAAGAGGG		This study
Var1R		GAAACTAATCTTAAGCGTTCC	895	This study
Var2F	2A	GGAACCATTGAAGTAAGG		This study
Var2R		CTTTCAGACCTAACATTTGAC	546	This study
Var3F	3, 4	GCTTCCAGATGCAAAAACG		This study
Var3R		GTGTTACTATCATGGCAAG	271	This study
Var4F	6	GTGAAGAATGTAGATGACC		This study
Var4R		CAATAACAAGTGCTAAGAC	1,086	This study
Var5F	7	CCCCATTGGAGGATATACGCAG		This study
Var5R		TGGGTAGTACGGTTCGTCAC	376	This study
Multiplex system 2				
Fg1F	1, 6, 7	GTCATGTGCTCTACCAATTG		This study
Fg1R		GATGGAGCTTATAACGTTC	1,481	This study
Fg2F	2, 2A, 3	GTCTTTCATACCATCCATG		This study
Fg2R		GTTCAACTGCTTATTATCG	1,040	This study
Fg3F	4, 5	GCTTGGCATGATGGTATG		This study
Fg3R		GAATAACATCACGTCCTCG	852	This study

### Comparative analysis of the *rgp* loci of S. thermophilus strains.

The genomic regions corresponding to the *rgp* locus (for the purpose of the analysis, all gene content between the 30S ribosomal subunit and a predicted transcriptional regulator, represented by *orf07010*_UCCSt50_ and *orf06885*_UCCSt50_, respectively) from 78 streptococcal strains, 5 representative strains from the UCC collection and 73 strains from the NCBI database for which whole-genome sequence data were available (see Table S7) were collated and compared using an all-against-all, bidirectional BLAST alignment (38) with a cutoff value of 0.0001 and a minimum standard of 50% identity across 50% of the amino acid sequences of the encoded gene products. The phylogenetic relationship of the *rgp* loci, based upon proteomic content, was established through the application of the Markov clustering (MCL) algorithm via the mclblastline pipeline v12-0678 (42). A hierarchical clustering (HCL) analysis was performed and viewed using the multiexperiment viewer (MeV) (43). *Rgp* genotypes were assigned based on previously defined groups (14, 16) and/or through the identification of genetically distinct groups via HCL analysis and comparative alignments.

### Development of a refined multiplex PCR for classification of S. thermophilus *rgp* loci.

Bioinformatic and comparative analyses of *rgp* loci of representative dairy streptococcal strains assessed in this study identified unique and group-specific genes within the region responsible for biosynthesis of the variable structure that formed the basis of a refined multiplex PCR system for an enhanced and more discriminatory classification of S. thermophilus strains (multiplex system 1). Romero and colleagues (15) furthermore identified that the *rgpF* gene is genotype specific; therefore, this gene formed the basis of a second mPCR system to rapidly assign a genotype based on the rhamnan backbone biosynthesis-associated region of the *rgp* cluster. A universal positive control was included for both systems, based on the highly conserved DNA primase-encoding gene, which is located adjacent to the *rgp* cluster. Multiplex systems 1 and 2 incorporated four and five primer pairs, respectively, including the control primer pair (Table 3). Each set was applied to 21 strains from the UCC collection, which had previously been evaluated by the established single-step mPCR system and representing Rgp groups 1 to 4 (14, 16), to verify their efficacy under the following PCR conditions: 98°C for 3 min followed by 35 cycles of 98°C for 10 s, 55.5°C for 30 s, and 72°C for 1 min 45 s, followed by a final extension at 72°C for 10 min. The ORFs identified as PCR targets are highlighted in Fig. 2, and the expected amplicon sizes are listed in Table 3.

### Data availability.

The genome sequences of S. thermophilus UCCSt10 (deposited under the strain name S. thermophilus CNRZ1151), UCCSt12 (deposited under the strain name S. thermophilus CNRZ385), UCCSt89, and UCCSt95 have been submitted to the GenBank database under accession numbers CP065483, CP065495, JANFMW000000000, and CP101646, respectively.
