# Supplementary material for: Bipartite rgp Locus Diversity in Streptococcus thermophilus Corresponds to Backbone and Side Chain Differences of Its Rhamnose-Containing Cell Wall Polysaccharide
Source: Appl Environ Microbiol. 2022 Nov 9;88(23):e01504-22. doi: 10.1128/aem.01504-22 (PMC9746298; doi:10.1128/aem.01504-22)
Supplement: Supplemental file 1 — Supplemental material. Download aem.01504-22-s0001.pdf, PDF file, 0.4 MB [file aem.01504-22-s0001.pdf]

Table S1. ^{1}H and ^{13}C NMR data (δ, ppm, D_2O, 40°C, 600 MHz) for the Rgp (PS) and its derivatives from *S. thermophilus* strain UCCSt95.

Sugar		H/C 1	H/C 2	H/C 3	H/C 4	H/C 5	H/C 6
α-Rha A PS-OX	H	5.22	4.02	4.03	3.63	3.84	1.37
	C	101.5	79.9	71.2	81.7	68.6	18.5
α-Rha B PS-OX	H	4.93	4.16	3.83	3.57	3.77	1.28
	C	103.3	71.0	78.5	72.8	70.4	17.8
β-GlcNAc C PS-OX	H	4.78	3.81	3.63	3.56	3.40	3.79; 3.90
	C	102.4	56.8	82.6	69.4	76.8	61.7
α-Rha D PS-OX	H	4.88	3.80	3.74	3.43	3.98	1.24
	C	102.4	71.8	71.3	73.1	70.0	17.7
α-Rha D OS	H	5.08	3.96	4.12	3.76	3.92	1.37
	C	99.2	79.7	71.1	81.8	68.9	18.1
α-Rha E OS	H	4.95	4.09	3.80	3.45	3.75	1.28
	C	103.4	71.2	71.2	73.2	70.4	17.9
anh-Man C OS	H	5.09	3.83	4.20	4.18	3.95	3.68; 3.77
	C	90.7	85.0	84.8	76.6	84.2	61.7
β-Gal F OS	H	4.66	3.54	3.67	3.92	3.68	3.76; 3.79
	C	104.8	72.8	74.0	69.8	76.4	62.1
α-Rha A DPS	H	5.20	4.09	3.96	3.52	3.84	1.33
	C	102.1	79.2	71.1	73.4	70.4	17.9
α-Rha B DPS	H	4.96	4.17	3.86	3.57	3.77	1.29
	C	103.3	71.1	78.7	72.8	70.4	17.8
α-Rha A PS	H	5.23	4.02	4.05	3.64	3.84	1.37
	C	101.6	80.2	71.2	81.7	68.6	18.4
α-Rha B PS	H	4.93	4.15	3.84	3.54	3.76	1.28
	C	103.3	71.1	78.7	72.8	70.2	17.9
β-GlcNAc C PS	H	4.80	3.79	3.61	3.56	3.40	3.79; 3.90
	C	102.4	57.1	82.8	69.5	76.7	61.6
α-Rha D PS	H	5.08	3.77	4.09	3.71	4.07	1.32
	C	100.6	81.4	71.0	81.9	68.8	17.9
α-Rha E PS	H	4.86	4.06	3.78	3.44	3.76	1.29
	C	103.7	71.3	71.3	73.3	70.2	17.9
β-Gal F PS	H	4.64	3.54	3.66	3.91	3.67	3.76; 3.79
	C	104.8	72.8	73.9	69.7	76.3	62.1

Table S2. ^{1}H and ^{13}C NMR data (δ, ppm, D_2O, 25°C, 600 MHz) for the amino polysaccharide PS-A (*N*-deacetylated Rgp) from *S. thermophilus* strain UCCSt89.

Sugar		H/C 1	H/C 2	H/C 3	H/C 4	H/C 5	H/C 6
α-Rha A	H	5.16	4.38	4.14	3.69	3.93	1.35
	C	102.2	78.2	71.0	72.2	70.4	18.0
α-Rha B	H	5.15	4.14	3.86	3.57	3.76	1.28
	C	102.5	82.0	78.8	72.8	70.5	17.8
β-GalN C	H	4.95	3.45	4.13	4.35	3.80	3.75-3.84
	C	102.1	53.7	80.1	68.2	76.2	62.1
β-Gal D	H	4.63	3.65	3.69	3.95	3.73	3.75-3.84
	C	105.1	71.9	73.7	69.7	76.5	62.1

Table S3. ^{1}H and ^{13}C NMR data (δ, ppm, D_2O, 25°C, 600 MHz) for the neutral polysaccharide PS-N (EPS) from *Streptococcus thermophilus* strain UCCSt89.

Sugar		H/C 1	H/C 2	H/C 3	H/C 4	H/C 5	H/C 6
α-Rha A	H	5.28	4.08	3.85	3.44	4.07	1.28
	C	101.8	71.4	71.3	73.2	69.7	17.7
α-Gal B	H	5.14	3.91	3.91	4.07	4.37	3.84; 4.04
	C	97.7	69.0	70.1	70.1	70.7	69.7
β-Gal*f* C	H	5.07	4.13	4.09	4.01	4.03	3.75; 4.06
	C	109.2	82.1	77.9	84.3	70.8	72.4
β-Rha D	H	4.92	4.35	3.76	3.53	3.46	1.36
	C	101.9	74.2	80.0	71.9	73.7	18.0
β-Glc E	H	4.53	3.30	3.50	3.43	3.61	3.71; 4.07
	C	104.1	74.2	76.7	70.8	76.1	68.0
β-Glc F	H	4.50	3.35	3.62	3.62	3.49	3.79; 3.95
	C	103.9	74.3	77.2	78.4	75.9	62.1
β-Gal G	H	4.45	3.54	3.66	3.95	3.89	3.91; 4.03
	C	104.3	72.0	73.8	69.8	75.0	70.6

Table S4. NMR data for *S. thermophilus* UCCSt12 cwps (Rgp) deamination product OS1 and its products of Smith degradation OS 2-19 and OS 2-20 (600 MHz, 25 °C, δ ppm).

Sugar		H/C 1	H/C 2	H/C 3	H/C 4	H/C 5	H/C 6
β-Gal E OS1	H	4.54	3.60	3.66	3.94	3.71	3.77; 3.77
	C	104.5	71.9	73.7	69.7	76.2	62.1
anhTal D OS1	H	5.08	3.93	4.40	4.41	4.15	3.78; 3.84
	C	91.0	83.0	82.5	73.2	82.3	61.2
Gral3d A OS 2-19	H	5.12	3.64	3.72; 3.85			
	C	90.3	81.1	60.4			
α-Rha B OS 2-19	H	5.04	4.30	3.93	3.52	3.85	1.29
	C	103.2	71.2	81.1	72.2	70.6	17.8
α-Rha C OS 2-19	H	4.97	4.10	3.88	3.54	3.98	1.28
	C	100.7	71.3	79.6	72.5	70.3	17.8
β-GalNAc D	H	4.66	3.95	3.75	3.94	3.68	3.77; 3.82
OS 2-19 and 2-20	C	104.5	53.8	72.2	69.0	76.2	62.3
Gral3d K OS 2-20	H	5.08	3.64	3.70; 3.85			
	C	90.4	81.2	60.7			
α-Rha L OS 2-20	H	5.20	4.07	3.79	3.45	3.70	1.27
	C	102.9	71.1	71.2	73.3	70.4	17.8
α-Rha M OS 2-20	H	5.05	4.26	3.95	3.54	3.96	1.28
	C	100.1	78.0	81.2	72.4	70.4	17.8

Table S5. NMR data for *S. thermophilus* UCCSt12 cwps (EPS) oxidation product OS 2-25 (500 MHz, 25 °C, δ ppm).

Sugar		H/C 1	H/C 2	H/C 3	H/C 4	H/C 5	H/C 6
α-Rha A	H	5.17	4.11	3.97	3.55	3.86	1.32
	C	100.8	77.4	70.6	73.0	70.7	17.8
α-Gal B	H	5.18	3.99	4.07	4.12	4.23	3.74; 3.74
	C	96.6	68.8	78.5	70.2	72.1	62.0
α-Glc C	H	5.01	3.55	3.78	3.45	4.04	3.78; 3.81
	C	98.9	72.5	73.9	70.7	73.1	62.2
β-Gal D	H	4.56	3.70	3.79	4.19	3.70	3.81; 3.81
	C	104.2	70.7	78.4	66.0	76.1	61.5
tetritol X	H	3.84	3.85	3.87	3.69; 3.81		
	C	62.1	82.6	72.0	63.6		

Table S6. Summary of the bioinformatic output of the UCCSt50 *rgp* locus

Locus tag	Predicted function	TMH
UCCSt50_7010	30s ribosomal protein S21	0
UCCSt50_7000	DNA primase	0
UCCSt50_6995	RNA polymerase sigma factor RpoD	0
UCCSt50_6990	Hypothetical protein	0
RgpI	Glycosyltransferase family 2 (GT2); Pfam00535; 77% identity to RgpI	2
UCCSt50_6980	DUF2142; Pfam09913 (PMT_2 family)	11
RmlD	dTDP-4-dehydrorhamnose reductase; dTDP-L-Rhamnose biosynthesis	0
ScbF	Wzx like flippase; polysaccharide transport. Pfam1943	12
ScbC	GT2; Pfam00535	0
ScbD	GT2; Pfam00535	0
ScbA	GT2; Pfam00535; 51.46% identity to WpsA of *L. lactis* NZ9000 (GacI homolog); UDP-GlcNAc:Und-P-GlcNAc transferase	0
ScbB	DUF2304; Pfam009913; predicted activator of *S. thermophilus* ScbA. Pfam10066	3
ScbE	GT2; Pfam00535	0
UCCSt50_6940	Glycosyltransferase - multidomain;	0
UCCSt50_6935	DUF2142; Pfam09913 (PMT_2 family)	9
RgpE	Glycosyltransferase family 4 (GT4); GT4_PimA-like	0
UCCSt50_6925a	hypothetical protein – interrupted	1
UCCSt50_6925b	hypothetical protein – interrupted	6
RgpA	DUF1972; GT4-like. Pfam09314	0
RgpB	GT2. Pfam00535	0
RgpC	ABC transporter permease. Pfam01061	6
RgpD	ABC transporter ATP binding. Pfam00005	0
RgpF	Pfam05045 - RpgF	0
UCCSt50_6895	DNA repair protein. Pfam04002	0
UCCSt50_6890	glutamine amidotransferase (class I). Pfam07722	0
UCCSt50_6885	Rex family transcriptional regulator. Pfam02629	0

Table S7: *S. thermophilus* strains used in this study

Strain	Source	Accession number	Strain	Source	Accession number
**UCCSt12*†**	UCC collection	CP065495	**StCNRZ760†**	UCC collection	N/A
**UCCSt50*†**	UCC collection	CP065477	**CNRZ887†**	UCC collection	N/A
**UCCSt89*†**	UCC collection	JANFMW000000000	**CNRZ1202†**	UCC collection	N/A
**UCCSt95*†**	UCC collection	CP101646	**CNRZ1066***	NCBI	N/A
**UCCSt10*†**	UCC collection	CP065483	**St_CIRM_16***	NCBI	NZ_LR822006
**UCCSt97†**	UCC collection	N/A	**St_CIRM_18***	NCBI	NZ_LR822008
**St4067†**	UCC collection	N/A	**St_CIRM_19***	NCBI	NZ_LR822009
**St4134†**	UCC collection	N/A	**St_CIRM_23***	NCBI	NZ_LR822011
**St4145†**	UCC collection	N/A	**St_CIRM_29***	NCBI	NZ_LR822010
**St4147**	UCC collection	N/A	**St_CIRM_30***	NCBI	NZ_LR822012
**St90728†**	UCC collection	N/A	**St_CIRM_32***	NCBI	NZ_LR822013
**StAVA116†**	UCC collection	N/A	**St_CIRM_36***	NCBI	NZ_LR822014
**StAVA1121†**	UCC collection	N/A	**St_CIRM_65***	NCBI	NZ_LR822015
**St4052†**	UCC collection	N/A	**St_CIRM_67***	NCBI	NZ_LR824002
**StMM20†**	UCC collection	N/A	**St_CIRM_336***	NCBI	NZ_LR822017
**StR1†**	UCC collection	N/A	**St_CIRM_368***	NCBI	NZ_LR822023
**St1A†**	UCC collection	N/A	**St_CIRM_1116***	NCBI	NZ_LR822039
**St128†**	UCC collection	N/A	**St_CIRM_1121***	NCBI	NZ_LR822037
**StCNRZ302†**	UCC collection	N/A	**St_CIRM_1125***	NCBI	NZ_LR822040
**St_CIRM_772***	NCBI	NZ_LR822019	**St_CIRM_1358***	NCBI	NZ_LR822042
**St_CIRM_956***	NCBI	NZ_LR822020	**St_CIRM_2101***	NCBI	NZ_LR822043
**St_CIRM_961***	NCBI	NZ_LR822025	**St_ST106***	NCBI	NZ_CP031881
**St_CIRM_967***	NCBI	NZ_LR822026	**St_ACA_DC2***	NCBI	NZ_LT604076
**St_CIRM_998***	NCBI	NZ_LR822027	**St_APC151***	NCBI	NZ_CP019935
**St_CIRM_1035***	NCBI	NZ_LR822029	**ST_ASCC1275***	NCBI	NZ_CP006819
**St_CIRM_1046***	NCBI	NZ_LR822030	**St_ATCC12958***	NCBI	NZ_CP038020
**St_CIRM_1047***	NCBI	NZ_LR822031	**St_B59671***	NCBI	NZ_CP022547

**St_CIRM_1048***	NCBI	NZ_LR822033	**St_CS5***	NCBI	NZ_CP028896
**St_CIRM_1049***	NCBI	NZ_LR822034	**St_CS8***	NCBI	NZ_CP016439
**St_CIRM_1050***	NCBI	NZ_LR822032	**St_CS9***	NCBI	NZ_CP030927
**St_CIRM_1051***	NCBI	NZ_LR822035	**St_CS18***	NCBI	NZ_CP030928
**St_CIRM_1055***	NCBI	NZ_LR822036	**St_NCTC12958**	NCBI	NZ_LS483339
**St_DGCC7710***	NCBI	NZ_CP025216	**St_ND03***	NCBI	NC_017563
**St_EPS***	NCBI	NZ_CP025400	**St_ND07***	NCBI	NZ_CP016394
**St_EU01***	NCBI	NZ_CP047191	**St_S9***	NCBI	NZ_CP013939
**St_GABA***	NCBI	NZ_CP025399	**St_SMQ-301**	NCBI	NZ_CP011217
**St_IDCC2201***	NCBI	NZ_CP035306	**St_ST3***	NCBI	NZ_CP017064
**St_JIM8232***	NCBI	NC_017581	**St_ST109***	NCBI	NZ_CP031545
**St_KLDS3***	NCBI	NZ_CP016877	**St_64987***	NCBI	NZ_CP049053
**St_CIRM_1122***	NCBI	NZ_LR822041	**St_TH982***	NCBI	NZ_CM003136
**St_KLDS_SM***	NCBI	NZ_CP016026	**St_TH985***	NCBI	NZ_CM003139
**St_LMD9***	NCBI	NC_008532	**St_TH1435***	NCBI	NZ_CM002369
**St_LMG18311***	NCBI	NC_006448	**St_TH1436***	NCBI	NZ_CM002370
**St_ M17PTZA496***	NCBI	NZ_CM002372	**St_TH1477***	NCBI	NZ_CM003135
**St_MNBMA01***	NCBI	NZ_CP012588	**St_TKP3A***	NCBI	NZ_CP045596
**St_MNBMA02***	NCBI	NZ_CP010999			
**St_MNZLW002***	NCBI	NC_017927			
**St_MTH17CL396***	NCBI	NZ_CM002371			
**St_N4L***	NCBI	NZ_LS974444			

*denotes strain included to HCL analysis

† denotes strain subject to Multiplex PCR system 1 and Multiplex PCR s

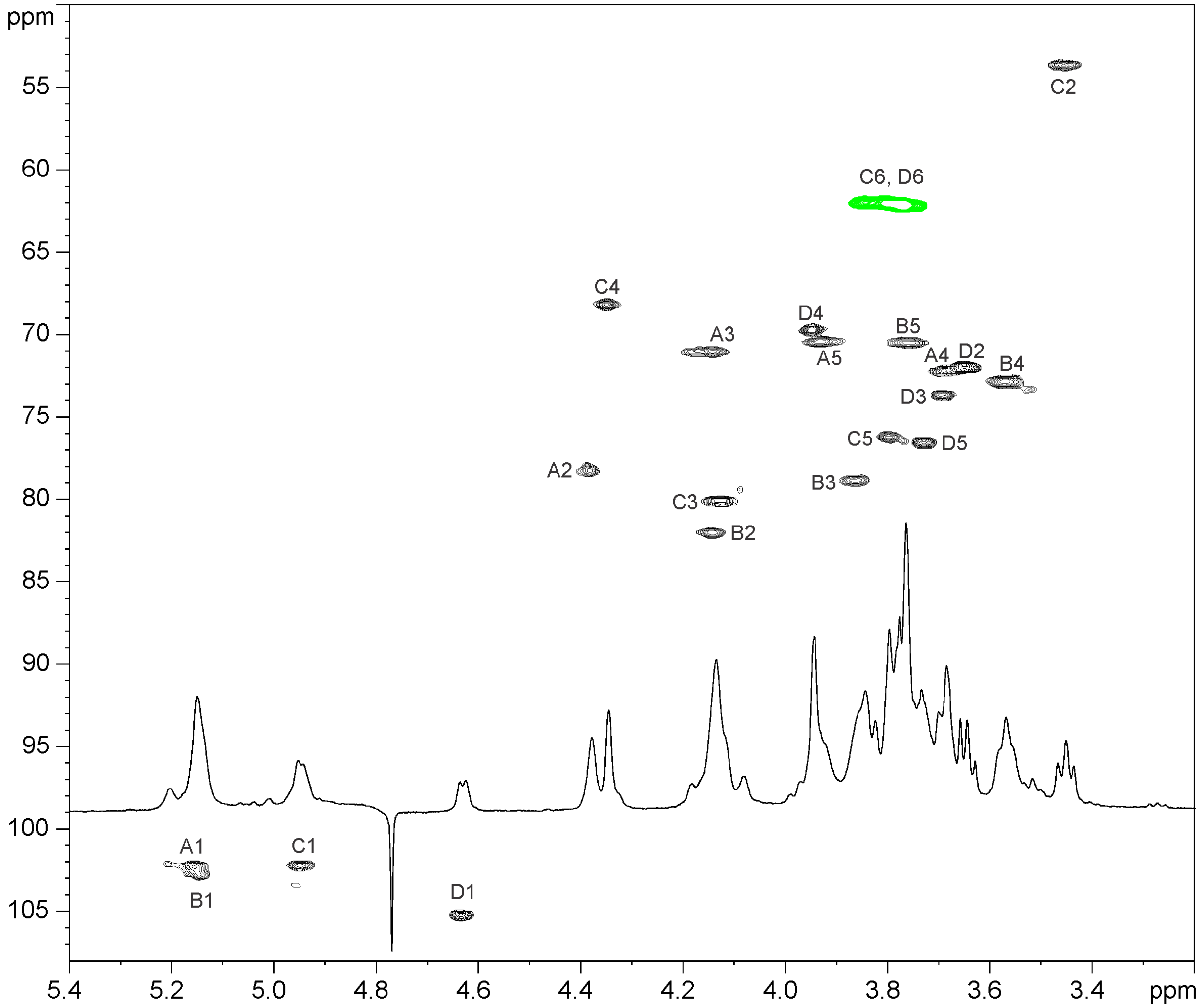

PS-A

```
        A          B
-2-α-Rha-3-α-Rha-
                   |2
  β-Gal-3-β-GalN
      D          C
```

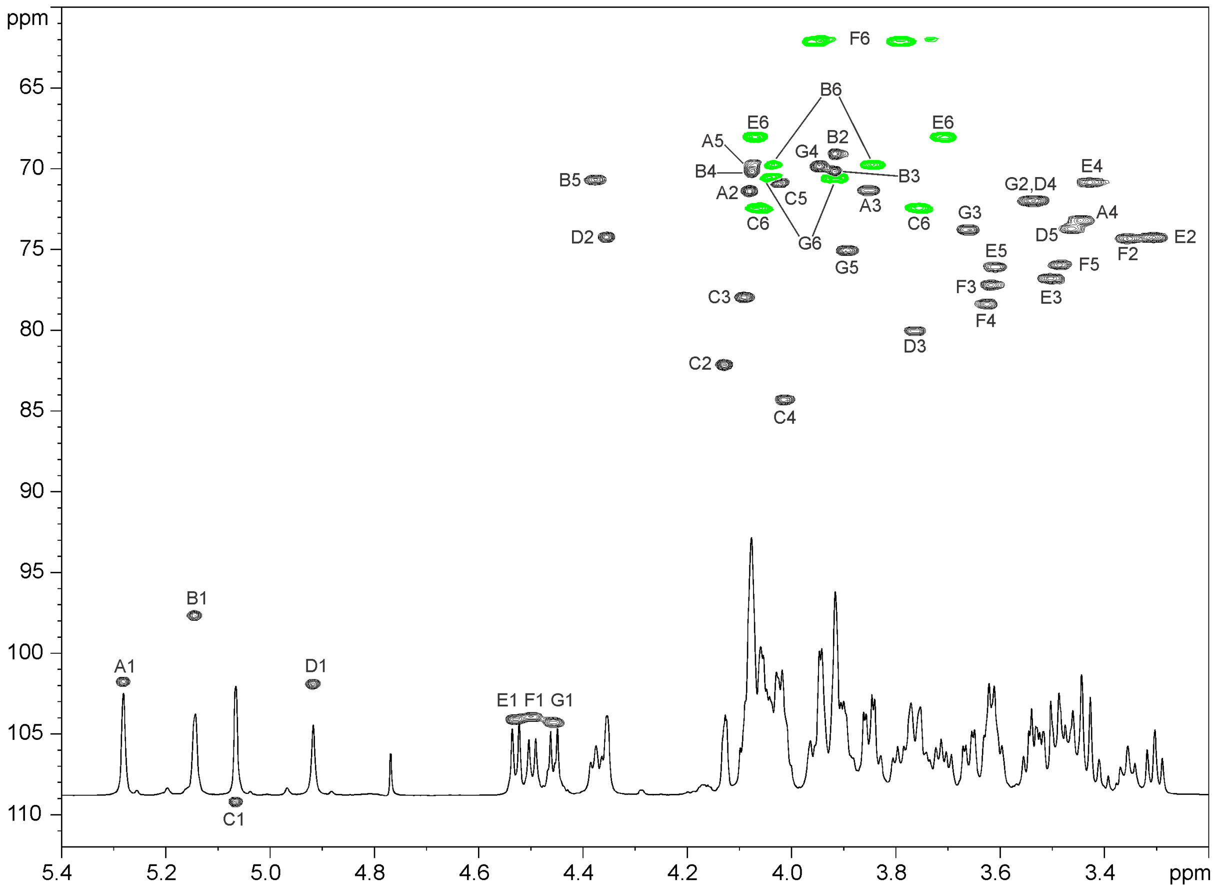


```
        B          D          F          C          E          G
-6-α-Gal-3-β-Rha-4-β-Glc-6-β-Galf-6-β-Glc-6-β-Gal-          PS-N
                    |2
                 α-Rha
                    A
```

Figure S1 ^{1}H-^{13}C-HSQC spectra of the *S. thermophilus* UCCSt89 cwps: the amino polysaccharide PS-A (*N*-deacetylated Rgp) and neutral polysaccharide PS-N (EPS). Spectra obtained in D_2O at 25 °C, 600 MHz.
